# Supplementary figures and images for: Two new species of Eleutherodactylus from western and central Mexico (Eleutherodactylus jamesdixoni sp. nov., Eleutherodactylus humboldti sp. nov.)
Source: PeerJ. 2023 Mar 8;11:e14985. doi: 10.7717/peerj.14985 (PMC10007972; doi:10.7717/peerj.14985)

*E. zeus* USNM 335740  
*E. symingtoni* No voucher

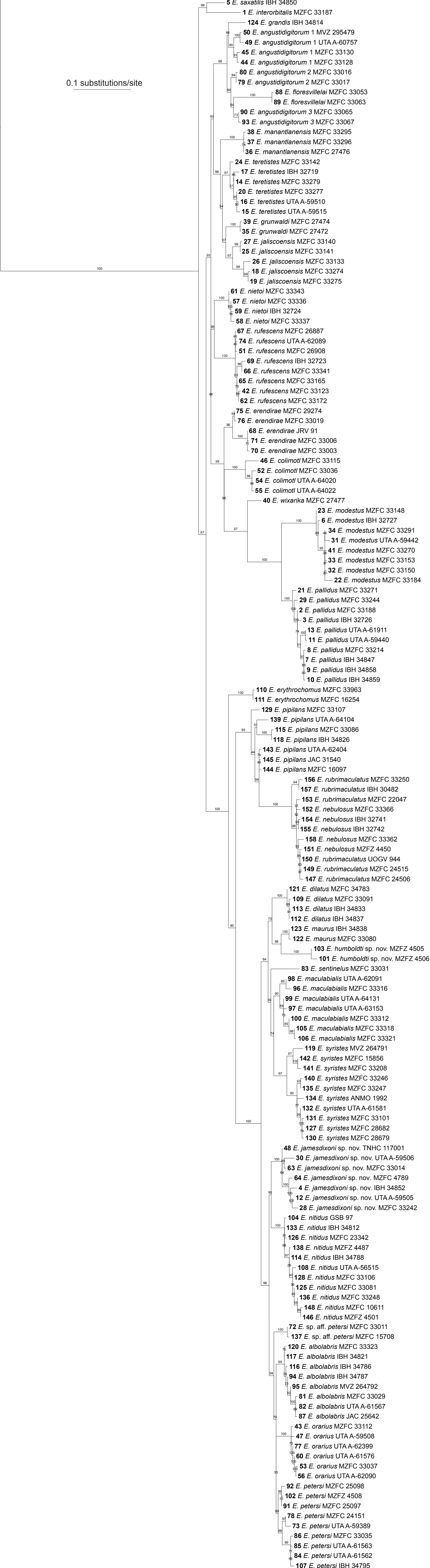

Supplement: Supplemental Information 7 — Numbers on nodes represent ultrafast bootstrap support values from 10,000 ultrafast bootstrap replicates. [file peerj-11-14985-s007.pdf]
