# Supplementary material for: Two new species of Eleutherodactylus from western and central Mexico (Eleutherodactylus jamesdixoni sp. nov., Eleutherodactylus humboldti sp. nov.)
Source: PeerJ. 2023 Mar 8;11:e14985. doi: 10.7717/peerj.14985 (PMC10007972; doi:10.7717/peerj.14985)
Supplement: Supplemental Information 8 — The character number (first column) refers to the position of the site in our alignment (Supplemental Data S2). [file peerj-11-14985-s008.docx]

Supplemental Table S2. Unambiguous synapomorphic characters and the change from ancestral to derived state of Eleutherodactylus species (or clades in which neither of the two species is monophyletic). The character number (first column) refers to the position of the site in our alignment (Supplemental Data S2).

*Eleutherodactylus interorbitalis*

159 T ==> C

164 C ==> T

165 A ==> G

205 T ==> C

257 G ==> A

279 C ==> T

340 A ==> G

529 C ==> T

*Eleutherodactylus saxatilis*

163 C ==> T

204 C ==> T

207 T ==> C

243 A ==> C

269 C ==> A

307 A ==> G

364 A ==> G

428 T ==> C

*Eleutherodactylus erendirae*

200 A ==> G

204 C ==> A

295 A ==> C

529 C ==> T

*Eleutherodactylus colimotl*

123 T ==> C

149 A ==> G

287 C ==> T

296 C ==> T

317 A ==> G

477 T ==> A

*Eleutherodactylus wixarika*

269 T ==> C

476 C ==> T

543 A ==> G

545 C ==> A

587 A ==> G

*Eleutherodactylus modestus*

146 C ==> T

160 T ==> A

164 C ==> T

257 G ==> A

295 G ==> C

343 A ==> T

367 T ==> C

401 A ==> T

428 T ==> C

474 C ==> T

486 C ==> A

496 G ==> A

*Eleutherodactylus pallidus*

204 C ==> T

205 T ==> C

288 C ==> T

394 T ==> C

406 C ==> T

478 A ==> G

527 T ==> C

*Eleutherodactylus nietoi*

146 C ==> T

165 A ==> G

257 G ==> A

281 C ==> A

*Eleutherodactylus rufescens*

29 A ==> G

271 C ==> A

272 T ==> A

273 T ==> C

274 A ==> C

287 C ==> T

343 A ==> T

359 A ==> C

378 A ==> G

529 C ==> T

*Eleutherodactylus grandis*

253 T ==> A

269 T ==> C

279 C ==> T

287 C ==> T

292 G ==> A

294 A ==> G

299 C ==> A

300 A ==> T

*Eleutherodactylus angustidigitorum 1*

147 A ==> C

206 T ==> C

368 T ==> C

*Eleutherodactylus angustidigitorum 2*

279 C ==> T

308 A ==> G

480 G ==> A

*Eleutherodactylus angustidigitorum 3*

148 A ==> G

287 C ==> T

*Eleutherodactylus floresvillelai*

131 T ==> C

205 T ==> C

206 T ==> A

207 T ==> C

273 T ==> C

277 T ==> C

281 C ==> T

290 T ==> C

291 C ==> T

309 T ==> C

317 G ==> A

367 C ==> T

402 T ==> C

406 C ==> T

483 T ==> C

547 C ==> T

*Eleutherodactylus manantlanensis*

131 T ==> C

274 A ==> C

278 A ==> C

290 T ==> C

299 C ==> T

360 C ==> T

380 A ==> G

477 T ==> C

497 T ==> C

*Eleutherodactylus teretistes*

95 G ==> A

277 C ==> T

292 G ==> A

*Eleutherodactylus grunwaldi*

58 T ==> C

146 C ==> T

281 C ==> T

359 A ==> T

380 A ==> G

529 C ==> T

587 A ==> G

*Eleutherodactylus jaliscoensis*

269 T ==> C

291 C ==> T

414 A ==> T

*Eleutherodactylus erythrochomus*

58 T ==> C

115 T ==> C

204 C ==> T

252 T ==> C

345 C ==> T

453 T ==> A

483 T ==> C

524 A ==> G

541 C ==> T

*Eleutherodactylus pipilans-rubrimaculatus-nebulosus*

271 C ==> T

291 C ==> A

292 G ==> A

360 C ==> T

*Eleutherodactylus sentinelus*

255 A ==> G

257 G ==> A

273 T ==> C

281 C ==> T

292 G ==> A

360 C ==> A

363 A ==> G

369 T ==> C

383 C ==> T

587 G ==> A

*Eleutherodactylus maculabialis*

316 C ==> T

486 C ==> T

*Eleutherodactylus humboldti* sp. nov.

147 A ==> G

204 C ==> T

241 C ==> T

243 A ==> T

256 T ==> C

279 C ==> A

281 C ==> T

292 G ==> A

295 A ==> G

342 T ==> C

*Eleutherodactylus maurus*

475 T ==> C

478 A ==> G

483 T ==> C

*Eleutherodactylus dilatus*

290 T ==> C

342 T ==> C

367 T ==> C

486 C ==> T

491 G ==> A

532 C ==> T

*Eleutherodactylus syristes*

103 C ==> T

204 C ==> T

279 C ==> T

291 C ==> T

309 T ==> C

316 C ==> T

*Eleutherodactylus albolabris*

176 C ==> T

359 A ==> C

*Eleutherodactylus* sp. aff*. petersi*

144 G ==> A

148 C ==> A

236 A ==> G

277 T ==> A

278 A ==> C

364 A ==> G

*Eleutherodactylus petersi*

268 T ==> C

*Eleutherodactylus orarius*

256 T ==> C

276 A ==> T

291 C ==> T

293 C ==> T

384 C ==> T

388 G ==> A

*Eleutherodactylus jamesdixoni* sp. nov.

204 C ==> T

291 C ==> A

360 C ==> A

394 C ==> A

443 T ==> C

*Eleutherodactylus nitidus*

37 A ==> G

131 C ==> T

268 T ==> C

296 C ==> T

402 T ==> C

428 T ==> A

475 T ==> C
